# Testing the Psychometric Properties of a Chinese Version of the Level of Expressed Emotion Scale

**DOI:** 10.1155/2014/905950

**Published:** 2014-01-06

**Authors:** Wai Tong Chien, Zenobia Chung-Yee Chan, Sally Wai-Chi Chan

**Affiliations:** ^1^School of Nursing, Faculty of Health and Social Sciences, The Hong Kong Polytechnic University, Hung Hom, Kowloon, Hong Kong; ^2^Alice Lee Centre for Nursing Studies, National University of Singapore, Singapore

## Abstract

This study tested the psychometric properties of a Chinese version of the level of expressed emotion scale in Hong Kong Chinese patients with severe mental illness and their family caregivers. First, the semantic equivalence with the original English version and test-retest reliability at 2-week interval of the Chinese version was examined. After that, the reproducibility, construct validity, and internal consistency of the Chinese version were tested. The Chinese version indicated good semantic equivalence with the English version (kappa values = 0.76–0.95 and ICC = 0.81–0.92), test-retest reliability (*r* = 0.89–0.95, *P* < 0.01), and internal consistency (Cronbach's **α** = 0.86–0.92). Among 262 patients with severe mental illness and their caregivers, the 50-item Chinese version had substantial loadings on one of the four factors identified (intrusiveness/hostility, attitude towards patient, tolerance, and emotional involvement), accounting for 71.8% of the total variance of expressed emotion. In confirmatory factor analysis, the identified four-factor model showed the best fit based on all fit indices (*χ* ^2^/df = 1.93, *P* = 0.75; AGFI = 0.96; TLI = 1.02; RMSEA = 0.031; WRMR = 0.78) to the collected data. The four-factor Chinese version also indicated a good concurrent validity with significant correlations with family functioning (*r* = −0.54) and family burden (*r* = 0.49) and a satisfactory reproducibility over six months (intraclass correlation coefficient of 0.90). The mean scores of the overall and subscale of the Chinese version in patients with unipolar disorder were higher than in other illness groups (schizophrenia, psychotic disorders, and bipolar disorder; *P* < 0.01). The Chinese version demonstrates sound psychometric properties to measure families' expressed emotion in Chinese patients with severe mental illness, which are found varied across countries.

## 1. Introduction

Since the pioneering work of Brown et al. in 1960s and Vaughn and Leff in 1970s [1, 2], expressed emotion (EE) has provided an index of the emotional climate of and attitude toward people with schizophrenia within their family environment. The EE refers to the amount of criticism, hostility, positive remarks, warmth, and emotional over-involvement expressed in family relationships, particularly among relatives of a mentally ill patient [2], and the concept and role of both positive and negative emotions and intrusive attitudes of family members in relation to schizophrenia care have effectively been evidenced in recent research. It is conclusive that patients with schizophrenia discharged to home environment with a high level of EE relapse at a much higher rate than those with low expressed emotion in family, and EE is considered an important predictor of the course and relapse of a few mental disorders such as depression and anxiety disorders and, subsequently, of a number of physical and psychiatric conditions, ranging from dementia to Parkinson's disease and diabetes [2, 3]. Of the five original proposed components, criticism, hostility, and emotional overinvolvement are shown to be the most predictive of patients' relapse from illness, particularly in schizophrenia and mood disorders [4, 5]. While the interactions between EE and patient outcomes are complex, recent studies have indicated that EE is closely correlated with patients' positive symptoms and adherence to medication and family's burden of care and functioning [4, 6].

While most studies on EE build on the “traditional” measurement by rating the attitudes and feelings expressed toward a patient by one of the main caregivers during the Camberwell Family Interview (CFI) [7], the widespread application of this assessment has been limited by the lengthy training and administration required, its complex scoring system, and the availability of a key relative. This has prompted the design of a less cumbersome alternative measure of EE for more feasible use in routine clinical settings. Magana et al. [7] have introduced the Five-Minute Speech Samples as a relatively brief assessment method; however, the drawbacks such as required involvement of a key relative and the complex scoring system still exist.

Martens and Addington [6] indicate that perceived criticism by depressed patients was more predictive of relapse in depressive disorder than the amount of criticism actually expressed by family members during the interview using the CFI. Therefore, recent literature suggested that patients themselves should be the focus of assessment in understanding their perceptions of the influential relationship with and attitudes of their families. Comments and emotions expressed by family caregivers may be perceived by their relative with mental illness as signs of love and care, or sometimes as coercive attempts to restore his/her desirable social behavior [8]. The 60-item level of expressed emotion scale (LEE) developed by Cole and Kazarian [9] is the only available valid instrument that addresses the importance of and increasing evidence on high validity of perceived EE by patients about their own behavior [2, 9]. It is a self-reported measure of a patient's perception of interactions with family during the previous one to three months and initially 25 psychologists generated 604 items based on four aspects of EE of family members, which included the level of intrusiveness, emotional responses to the illness, negative attitude towards illness, and level of tolerance and expectations concerning the patient. A panel of psychologists and psychiatrists rated and agreed that 60 of the items reflected the construct of patients' perceived EE in their family, which possessed the highest item-scale correlations, the lowest social desirability loadings, and the highest content relevance and appropriateness of their respective dimensions (subscales) of EE [10, 11]. Feasibility and accuracy of asking about patients' perceptions of EE with careful consideration to the influence of psychotic symptoms were found satisfactory [2, 6, 9].

In addition, Cole and Kazarian [9] also reported that the relationship between the LEE and CFI was satisfactory in total score; however, the correlations between the four subscales (intrusiveness, illness attitude, expectancy/tolerance, and emotional responses) were slightly significant (*P* = 0.04-0.05). As suggested by Gerlma and Hale III [2], the findings indicate that patients and family members may show differential focus on the correlates of EE. The results are encouraging and support the value of self-report measure in evaluating the affective environment of patients with schizophrenia, and in need of replication to other samples [2, 10]. With such encouraging results, it is also important to further explore whether high expressed emotion perceived by patients can be predictive of symptom exacerbations and relapse from schizophrenia and other severe mental illnesses at least six months later, as measured with the “traditional” measurements from a main caregiver [4, 6, 8].

The family's EE of people with mental illness has not been adequately considered from a cultural perspective, particularly in Chinese populations [12]. The components of EE and their relative intensities are most likely to vary across cultures. For example, in India, emotional involvement is the norm and if a carer does not show much emotional involvement, it is seen as lack of care for the ill relative [13]. In Chinese population, Li and Arthur [12] and Philips et al. [14] found that over 40% of Chinese family members of patients with schizophrenia were rated as showing high EE. They also observed that there was a significant increase in the relative risk of illness relapse for the Chinese patients with high EE, when compared to those in low EE families. This finding may be due to less impact on the Chinese patients of a high EE relative, or the role of individual family members better seen by these patients as a protective factor [15].

In Hong Kong and other Chinese communities, families are dominated by traditional Confucian and Buddhist principles. The strong values of interdependence and obligation of family care are dominant in Chinese families, indicating great emphases on withholding criticism and instilling hope among family members [16]. In addition, with these specific traditional beliefs and values, Chinese families often indicate strong interdependence and collective actions, high acceptance of social roles, and less expressive and open communication with family members, colleagues, and friends but, on the other hand, more preferring practical assistance in life events, for example, assisting in family chores and financial difficulties [17]. There is also a belief in keeping secret about something unfortunate or degrading the family name and a strong emphasis on instilling hope among family members [12, 17]. There may be significant cultural influences affecting the family's attitude and feelings towards mentally ill patients.

Therefore, LEE scale had been translated into Chinese language by the research team and its face and content validity and internal consistency were tested in a convenience sample of Chinese patients primarily diagnosed with schizophrenia in Hong Kong [11]. The Chinese translated version of the LEE scale indicated a high level of item and overall scale equivalence with the original English version (i.e., kappa values of items ranged from 0.70 to 0.90 and intraclass correlation coefficients between the two versions were 0.93). The Chinese version was then reduced from 60 to 52 items, taking account of two important results of testing: (a) four items having exceptionally low item-total correlations (i.e., range: 0.15–0.20) and an increase of 0.10 of Cronbach's alpha after deletion and (b) another four items with very low factor loadings (0.12–0.18) in the finalized four-factor solution in exploratory factor analysis with varimax rotation [18]. The resulting four factors of the 52-item Chinese version of LEE scale accounted for around 70% of the total scale variance, were significantly and moderately correlated with LEE overall scale and themselves (0.52–0.60, *P* < 0.01), and were significantly correlated with family and patient functioning scales (0.34–0.50 and 0.42–0.51, resp., and *P* < 0.05). In view of better understanding about the influence of family environment on Chinese mentally ill patients and preparing for comparisons of its findings of these patients' perceived EE across Chinese and other cultures, the present study examined the psychometric properties of the Chinese version of LEE scale in a more larger-sized, diverse sample with severe mental illness in Hong Kong.

## 2. Methods and Materials

The purpose of this study was to test the reliability and validity of a Chinese version of LEE scale and to identify the level of expressed emotion in families of outpatients with severe mental illness (SMI) in two-regional psychiatric outpatient clinics in Hong Kong. The patients with SMIs were those with a primary clinical diagnosis of schizophrenia and other psychotic disorders, mood disorders, and personality disorders according to the criteria of Diagnostic and Statistical Manual of Mental Disorders, 4th edition (DSM-IV), usually presenting a few psychotic symptoms [19]. The objectives of the study were:

1. to examine the equivalence between the original English and translated Chinese version;
2. to assess the test-retest reliability, internal consistency, reproducibility, and construct validity of the Chinese version;
3. to identify the level of patients' perceived expressed emotion in the Chinese families of patients with SMI and compare between illness subgroups.

## 3. Study Design

In the first phase of the study, the semantic equivalence of the original English and translated Chinese versions and test-retest reliability of the Chinese version were examined. In the second phase, the recruited SMI patients and their family caregivers were asked to complete a set of questionnaires twice over six months. These data were used to examine the internal consistency, reproducibility, responsiveness, and construct validity of the LEE scale and to identify the SMI patients' perceived EE in their families. For testing reproducibility of the LEE scale, the study subjects (patient-caregiver dyads) had to complete the questionnaire twice over the 6-month interval in order to assess its stability to detect the patients' mental state and/or family functioning.

## 4. Subjects and Setting

All the study subjects were recruited from two-regional psychiatric outpatient clinics in Kowloon and New Territories Hospital Clusters of Hospital Authority in which there were about 5,000 outpatients with various kinds of mental disorders being served (i.e., 30% of total psychiatric outpatients in Hong Kong) [20]. In the first phase of the study, a convenience sample of 40 patients with SMI in the clinic under study, who were proficient in English comprehension, was asked to complete both versions of the LEE scale for testing the semantic equivalence of the two versions. For assessing the test-retest reliability, another convenience sample of 40 patients with SMI was asked to complete the Chinese version twice with a two-week interval [18]. Those patients who participated in the first phase would be excluded from Phase 2 of the study.

In Phase 2, a cross-sectional descriptive survey was conducted among patients with SMI in the two-outpatient clinics under study. There were a total of about 2,500 outpatients with a primary diagnosis of SMI receiving follow-up treatment at the clinics [20]. A convenience sample of about 350 Chinese outpatients with SMI and one of each of their main family caregivers were invited to participate from the patient lists of the two clinics. As suggested by Stevens [18], at least five subjects per item should be required for factor analyses. This sample size also allowed a ±0.05 sampling error with 95% confidence level, achieving a power of 0.80 in the study, taking account of a potential nonresponse rate of around 20% [18, 21]. Those who met the study criteria described below were selected from the patient list and asked for consent to participate in the study by a research assistant (RA).

The inclusion criteria for sampling Chinese patients in the clinic were those who were (a) at age 18 or above and living with one or more family members over the last three months; (b) primarily diagnosed by attending psychiatrists with one type of the SMIs, according to the criteria of the DSM-IV [19]; and (c) able to understand Chinese/Mandarin and complete the questionnaire. Each main family caregiver referred to one of the family members who was responsible for most of the daily care for the patient and was subjected and agreed upon by the patient as his/her key carer in family. They also had to satisfy the following inclusion criteria: (a) at age 18 or above; (b) living with and caring for the patient with SMI over the past three months; and (c) being able to understand and read Chinese language.

However, those patients who suffered comorbidity of any other mental illnesses such as mental retardation and learning disability and/or chronic physical illness, who were mentally unstable as recently reported by their attending psychiatrist, or who had been discharged from a psychiatric (inpatient) hospital/unit within the past one month, were excluded. Those caregivers who themselves suffered from any chronic medical disease, mental illness, and/or cognitive impairment were also excluded.

## 5. Instruments

Seven research instruments were used in Phase 2 of the study and they are described as below.

*Level of Expressed Emotion (LEE) Scale (Client Version).* The 60-item LEE scale was designed by Cole and Kazarian [9] and translated into Chinese language by the research team with satisfactory content validity and internal consistency [11]. It is a self-reported measure of patient's perceptions of the level of expressed emotion in family interactions during the past 3 months. The scale consists of 4 a priori subscales-intrusiveness, attitude versus illness, expectancy/tolerance, and emotional response to illness, each comprising 15 items and requiring 4-point Likert-type responses: 1—“not true,” 2—“more or less untrue,” 3—“more or less true,” and 4—“true.” According to Chien and Chan [11], internal consistency of the Chinese version and its four subscales are high (Cronbach's *α* = 0.95 for the scale and 0.84–0.89 for subscales). According to Cole and Kazarian [9], test-retest reliabilities of the original and Chinese versions were satisfactory (*r* = 0.82 and 0.88, resp., and *P* < 0.01). Both versions also showed significant correlations with the family relationship and functioning scales [9, 11].

*Family Assessment Device (FAD). *The FAD developed by Epstein et al. [22] was used to assess multiple dimensions of family functioning among patients with schizophrenia and other mental illness. It consists of 60 items to measure family functioning in a 4-point Likert scale (from 1 = “strongly disagree” to 4 = “strongly agree”). There are seven subscales and four of them can be used to test their relationships with four dimensions of the LEE, namely, communication, affective responsiveness, affective involvement, and behavioral control. The Chinese version of the FAD demonstrates adequate content validity, high interrater reliability (ICC for overall scale was 0.85), and minimal social desirability effects in Chinese people with schizophrenia [11]. Chien and Chan [11] also reported that Cronbach's alpha was 0.97 for overall scale and ranged from 0.68 to 0.92 for subscales. Total score ranged from 4 to 28, a higher score reflects poorer family functioning; and the cut-off scores between healthy and unhealthy families ranged from 13 to 15.

*Family Burden Interview Schedule (FBIS).* The FBIS is a 25-item semistructured interview schedule designed by Pai and Kapur [23] to assess the burden of care experienced by families of a patient with schizophrenia living in the community. It consists of six domains of perceived burden (2–6 items in each domain), including effects on family finance, routine, leisure, interactions, physical health and mental health. The items are rated in a three-point Likert scale (0 = “no burden,” 1 = “moderate burden” and 2 = “severe burden.”). The total scores range from 0 to 50, with a higher score indicating higher burden of care. Interrater reliability for items was reported to be between 0.87 to 0.99, when being rated by mental health professionals and families [23]. Significant correlations with clinical psychopathology and social dysfunction in the patients were also reported. It was translated into Chinese by the research team and indicated high levels of equivalence with the original English version (intraclass correlations of 0.87 for overall scale and 0.80–0.89 for domains) [11]. The scale also demonstrated a high internal consistency (Cronbach's *α* = 0.87 for overall scale and 0.78–0.88 for domains) and an adequate test-retest response stability (*r* = 0.83–0.92 for the scale and its domains).

*Brief Psychiatric Rating Scale (BPRS). *The BPRS developed by Overall and Gorham [24] consists of 18 global, clinically familiar, symptom and behavior constructs that span much of the range of manifest psychopathology and are used effectively in clinical and research areas over the world for a few decades. The interviewer/assessor will rate each item of psychiatric symptom in a 7-point Likert scale (0—not present to 6—extremely severe). It demonstrated satisfactory reliability and validity in different psychiatric patient populations [24, 25]. Eight items of the BPRS (from thought disturbance and disorganization subscales, including item 4—“conceptual disorganization,” item 7—“mannerisms & posturing,” item 8—“grandiosity,” item 11—“suspiciousness,” item 12—“hallucinatory behavior,” item 14—“uncooperativeness,” item 15—“unusual thought content,” and item 18—”psychomotor excitement”) were used to assess the severity of positive symptoms [25, 26].

*Beck's Depression Inventory-II (BDI-II). *The Chinese version of Beck Depression Inventory is a 21-item self-reporting unidimensional measure of depression in terms of cognitive-affective and somatic dimensions. Studies have found satisfactory internal consistency reliability of BDI-II scores for both clinical and community care samples. The Chinese scale has been validated for use with both adults and adolescents [27] and items are rated on a 4-point Likert scale (from 0 = “nil specific problem” to 3 = “serious problem/difficulty”). Lower scores on the scale the better is the mood state of the patient (i.e., 0–10 = “mild” and 31–63 = “severe” depression) [27].

*Beck's Anxiety Inventory (BAI). *The Chinese version of Beck Anxiety Inventory is a 21-item self-reporting, uni-dimensional measure of anxiety, assessing its subjective, somatic and panic-related symptoms. Its suitability for use with adults had been confirmed, with an acceptable reliability and validity in different patient populations in Hong Kong and other Chinese people [28]. Items are rated on a 4-point Likert scale, ranging from 0 = “absence of symptoms” to 3 = “severe but barely acceptable”. The scores ranging between 0–21 and 42–63 indicate mild and severe anxiety state, respectively.

*Demographic Data Sheet. *A demographic data sheet was designed by the researchers and attached at the end page of the above questionnaires. This sheet included questions about the patient's age, gender, educational level, duration of illnesses, number of family members living with patient, average contact hours/week with primary caregiver, medication use, and length of rehospitalization in the past three months. It also included questions about the caregiver's age, gender, education level, relationship with patient, and monthly household income.

## 6. Data Collection Procedure

Ethical approval and permission to access patients' clinical information were obtained from the Research Ethics Committee of the hospital governing the clinics and The Hong Kong Polytechnic University. Patients' attending psychiatrists will be informed. Both patients' and family caregivers' written consents were obtained with full explanation of the study during subject recruitment. In Phase 1 for testing semantic equivalence of the Chinese and original English versions, a convenience sample of 40 patients with SMI was asked to complete both versions of the LEE scale. To minimize the effect of the sequence of questionnaire administration, one-half of respondents were given the Chinese version first and then the English version, and vice versa. Another convenience sample of 40 patients with SMI was also asked to complete the Chinese version twice with a 2-week interval for assessing the test-retest reliability [18].

In phase 2, data were collected over a period of about six months at the clinics under study. Eligible patients were invited to participate in the study by the trained research assistant (RA) during their follow-up consultations in the clinics. With full explanation of the research purpose and procedure, written consent was obtained from both the patients and their family caregivers. Each patient completed the self-report questionnaire (Chinese version of LEE scale and demographic data sheet) individually in an interview room of the clinics and returned it in a sealed envelope to the RA. It lasted about 15 minutes. When attending psychiatric consultation, the psychiatrist used the BPRS, BDI-II, and BAI to assess the individual patients' psychiatric symptoms (psychotic, depressive, and anxiety symptoms).

After the informed consent was obtained, the patients' family caregivers were interviewed in the clinic or via phone at home, using the FBIS and FAD by one RA and the interviews lasted about 15–20 minutes. Six months later, the patients and their family caregivers also completed the similar set of questionnaires when attending the clinics and during home visit or phone call, respectively.

## 7. Data Analysis

Descriptive and inferential statistics were employed on the data in the LEE scale and other measures, using the IBM SPSS for Windows version 20.0. The item equivalence between the Chinese and English version of the LEE scale was evaluated using weighted kappa, where a kappa of >0.4 indicated a satisfactory level of agreement on the translation of an item [18]. The equivalences between the subscales and the total scores of the two versions were assessed by intraclass correlation coefficient (ICC), using one-way ANOVA test. Pearson's product-moment correlation test was used to evaluate the test-retest reliability of the LEE at two-week interval. Internal consistency of the LEE scale was investigated using Cronbach's alpha coefficients, indicating the homogeneity of its construct.

Data from those patients who reported no major changes in both the severity of positive symptoms and family functioning between the first and second test over six months were used to assess the reproducibility of the LEE. ICCs were calculated using random effects one-way analysis of variance, whereas ICC = 0.7 or above represented satisfactory reproducibility [29].

Construct validity was established by (a) testing the correlations between the LEE scale and other measures with relevant theoretical constructs, including FAD and FBIS (i.e., *r* ≥ 0.5 would be reasonably intercorrelated), using Pearson's correlation test and (b) using an exploratory factor analysis (principal components analysis, with eigenvalue and Catell's scree test for determining appropriate number of components, and the varimax rotation for generating more interpretable factor solution) [18]. Bartlett's test of sphericity (*P* < 0.05) and the Kaiser-Meyer-Oklin measure of sampling adequacy (KMO index > 0.6) were used to assess the factorability of the data. Confirmatory factor analysis was then conducted to conclude the factor solution as explained by the scale items, using the LISREL 9.1 software [30]. After confirmatory factor analysis, the levels of patients' perceived EE (and all factor/subscale scores of LEE scale) were summarized to present the perceived family attitude and emotional involvement in the patients with SMI. Comparisons between the subgroups of SMI (schizophrenia, psychotic disorders, bipolar disorder, and unipolar depression) were performed to examine any significant differences of the levels of EE between psychiatric diagnoses.

## 8. Results of Phase 1

### 8.1. Characteristics of Participants

Two convenience samples of 40 patients with SMI (mainly 45% schizophrenia and 25% unipolar disorder) were recruited from the clinic, one group for equivalence testing and another group for test-retest reliability testing. The refusal rates were 13% and 15%, respectively, mainly due to time constraint and unwillingness to expose their mental condition. The mean values and ranges of age of two groups of 40 patients recruited were similar (M = 27.68, SD = 9.23, and range 22–40 and M = 27.03 years, SD = 9.68, and range 21–39, resp.). About two-thirds of them were male (*n* = 26 and 27) and completed secondary school or above education (*n* = 27 and 28). Their average durations of illness were 4.5 (SD = 2.8) and 5.1 years (SD = 3.1, both ranges: 1–9). Average lengths of rehospitalizations in the past three months were 7.1 (SD = 4.9) and 6.8 (SD = 4.5) days and their dosages of psychiatric medication were in moderate levels [11, 19].

### 8.2. Equivalence of the Chinese and English Versions of LEE Scale

The 52-item Chinese version of LEE scale indicated substantial agreement and thus good semantic equivalence with the original English version of LEE scale, in terms of both items and total scale [18]. Forty-seven items had a kappa >0.85 (i.e., range: 0.86–0.95) and the remaining five items had kappa values between 0.76 and 0.82 (i.e., items 20, 27, 37, 48, and 50). The intraclass correlation coefficients between the two versions were 0.90 (*P* = 0.01) for the total scale and from 0.81 to 0.92 for the four subscales (based on the previous exploratory factors analyses by the research team [11]). Only very minor amendments on the key terms/wordings of a few items were made (e.g., “realistic *領悟*” in item 27 changed to “realize *認識到*” and “Flies off the handle *狂怒* in item 48 to an ordinary, layman term “throws temper *發脾氣*”).

### 8.3. Test-Retest Reliability and Internal Consistency

Test-retest reliability coefficients for the Chinese version of LEE scale over the 2-week interval were *r* = 0.92 for the total scale (*P* = 0.01) and from 0.89 to 0.95 for the four subscales (*P* = 0.01–0.008). These results revealed that the Chinese version demonstrated a high stability of responses to the items over 2 weeks, and thus represented a high level of test-retest reliability. In addition, Cronbach's alpha coefficient of the Chinese version was 0.88 for the overall scale, indicating very satisfactory internal consistency of items to measure EE. The internal consistencies of the Chinese version and its subscales, together with the two measures with theoretical relevant construct, were also calculated and confirmed after factor analyses, thus presenting in later part of this report.

## 9. Results of Phase 2

### 9.1. Characteristics of Participants

Three hundred and twenty-five patients and/or their family caregivers were invited to participate in this study and the response rate was 82.0% (*n* = 267). Fifty-nine of them refused to participate. The main reasons of their refusals were as follows: without interest to participate (*n* = 28), too busy and lack of time to complete the questionnaires (*n* = 22), and unable to understand some items of the questionnaires (*n* = 9). Among the 267 voluntary participants, five patients and/or their caregivers did not complete all the questionnaires used, leaving 5–7 items unanswered, and these incomplete questionnaires were discarded for data analysis. The sociodemographic and clinical characteristics of 262 patients with SMI and one of each of their family caregivers who were finally included in the data analysis are summarized in Tables 1 and 2, respectively.

The final sample of 262 patients were mainly diagnosed with schizophrenia/psychotic disorders (*n* = 168, 64.1%) and mood disorders (bipolar or unipolar disorders *n* = 60, 22.8%). Their ages ranged from 19 to 40 years (M = 29.12, SD = 10.05), and about two-thirds were male (*n* = 160, 61.1%). Duration of their mental illness ranged from 12 to 98 months (M = 35.21, SD = 14.25), and the psychiatric medications used mainly included conventional and/or atypical antipsychotics (66.1%), antidepressants (19.1%), or both (7.6%). Average number and lengths of psychiatric hospitalizations during the previous three months were 0.40 times (SD = 0.29) and 8.12 days (SD = 4.11), respectively. Average contact time between the patients and their main caregivers was 30.40 hours per week (SD = 9.54). The results of Chi-square or independent sample *t* (two-tailed) test indicated that there were no significant differences in all patients' characteristics between the respondents and those who refused to participate (*P* > 0.30), except for the average number of rehospitalizations in the past three months (i.e., *t* = 3.38, df = 320, and *P* = 0.05; those who refused to participate had a significantly higher readmission rate than the respondents).

Their family caregivers were mainly parent (*n* = 98, 37.4%) and spouse (*n* = 63, 24.0%) and had secondary school education or above (*n* = 214, 81.7%). Their ages ranged from 21 to 67 years (M = 42.58, SD = 10.82). Two-thirds of them were female (*n* = 158, 60.3%) and had monthly household income of HK dollars 10,000–30,000 (*n* = 186, 71.0%) ranging from HK dollars 8,000 to 52,000. Similar comparisons between these caregivers' characteristics and those (i.e., 500 family caregivers of patients with schizophrenia, psychotic disorders and mood disorders) in the outpatient clinic using Chi-square or independent sample *t*-test were performed and the results were nonsignificant in all characteristics (*P* > 0.10).

### 9.2. Construct Validity and Exploratory Factor Analysis

Principal components analysis was conducted to identify the plausible underlying structures of the Chinese version of LEE scale. All corrected item-total correlations were positive with 49 out of 52 items falling within the range of 0.30–0.70. Only three items fell below the 0.30 criterion of adequate correlation with the total scale, including “understands my limitations” (item 23, *r* = 0.24), “can cope well with stress” (item 38, *r* = 0.23), and “is understanding if I make mistakes” (item 40, *r* = 0.25). However, the Cronbach's alpha for the overall scale was only increased by 0.05 when three items were deleted and thus they were not excluded from exploratory factor analysis.

The Kaiser-Meyer-Oklin value was 0.89 (i.e., >0.60), and Barlett's test of sphericity (0.79) reached a statistical significance (*P* = 0.10), thus supporting its factorability [18]. Results of principal components analysis indicated that there were four components (intrusiveness/hostility, attitude towards patient, tolerance, and emotional involvement) with eigenvalues greater than 1.0 in the unrotated matrix. By viewing the Catell's scree test, all four components were retained with 50 of 52 items, meeting the criterion of factor loading of 0.40 or above [18]. Only two items with factor loading of 0.17 and 0.19 were deleted from item rotation, including “Doesn't ask a lot of personal questions” and “Expects the same level of effort from me, even if I do not feel well”, respectively.

Varimax rotation was also performed to make the factors generally more interpretable. After rotation, each factor had high loading and all 50 items had substantial loadings (>0.40) on only one factor, except “Can cope well with stress” (item 46), as shown in Table 3. Item 46 was loaded into two factors, including “tolerance” (factor loading = 0.40) and “emotional involvement” (factor loading = 0.45). By interpreting its meaning and a higher indicated loading, it would only be counted in the identified factor “emotional involvement.” Factors derived from a rotated matrix were generally more interpretable because each factor tended to load high on a smaller number of items and low, or very low, on the other items using the rotation [18]. The total scale variance, indicated by the four factors, was 71.8% and the finalized 50 items and their factor solution (12 items for “Intrusiveness/Hostility”, 13 items for “attitude towards patient,” 12 items for “tolerance,” and 13 items for “emotional involvement”) were brought forward to undergo confirmatory factor analysis.

### 9.3. Construct Validity—Confirmatory Factor Analysis

The confirmatory factor analysis, after specifying the a priori factors, sought to optimally match the observed and theoretical factor structures for a given data set in order to determine the “goodness of fit” of the predetermined factor model with maximum likelihood. Three models were to be tested using LISREL version 9.1 for Windows [30], including the two-factor model suggested by the original authors Cole and Kazarian [9], the three-factor structure suggested by Gerlsma et al. [2] in their Dutch version and the four-factor model found in this research (i.e., the above results of exploratory factor analysis and very similar model by the research team in 2009 [11]). Modification indices specified the expected reduction in the overall *χ* ^2^ statistic by relaxing the constraints of no correlation or covariance between factors, as all factors being tested were hypothesized to capture the construct of EE in family environment [9, 11, 18]. A summary of the fit indices of the three-hypothesized models of the Chinese version of LEE scale with both uncorrelated and correlated factors is shown in Table 4.

As indicated in Table 4, the first model with correlated factors identified from the exploratory factor analysis in this study appears to fit the data very well. The four-factor model with paths between all factors showed very good fit based on all fit indices (*χ* ^2^/df = 1.93, *P* = 0.75; AGFI = 0.96; TLI = 1.02; RMSEA = 0.031; WRMR = 0.78) and was much better than the other two-factor or three-factor models. Critical ratios for the regression weights were all greater than 2.0, indicating that each item made a statistically significant contribution at the 0.05 level to its associated factor. Model modification indices for the four-factor model also pointed to good fit, especially with additional paths drawn between the four factors themselves. The second two-factor model proposed by the original authors was only an acceptable fit (*χ* ^2^/df = 2.02, *P* = 0.58; AGFI = 0.90; TLI = 0.95; RMSEA = 0.050; WRMR = 0.89) but a better fit than the three-factor model proposed by Gerlsma et al. [31] for their Dutch version.

**Figure 1:**
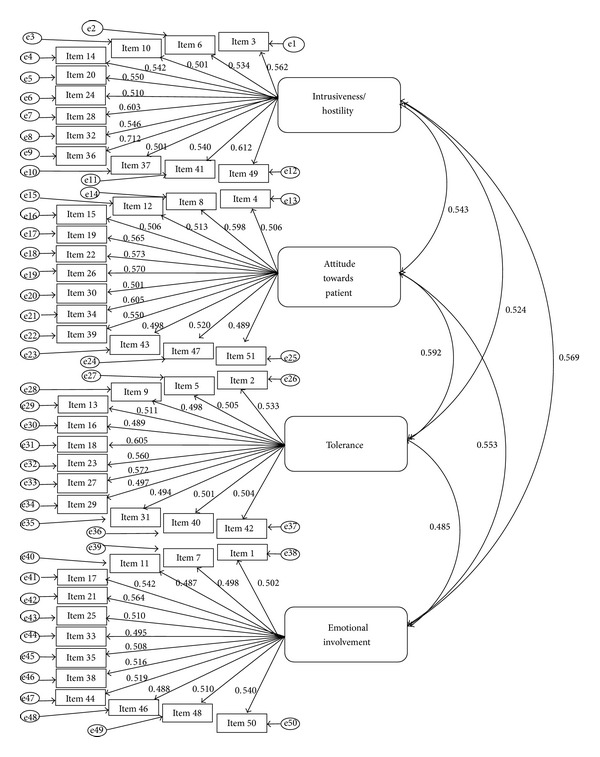
Path diagram for the four-factor model with standardized parameter estimates.

Path diagram of the best fit four-factor model is also presented in Figure 1. The diagram shows that the correlations between each of the four factors and their corresponding items ranged from 0.49 to 0.71, indicating a medium to medium-large associations or effects to each factor and thus the EE construct. The correlations between the four factors ranged from 0.49 to 0.59, again indicating moderate inter-relationships between them.

### 9.4. Internal Consistency of the Four-Factor Chinese Version of LEE Scale

With the above confirmed four-factor model, the Cronbach's alpha coefficients of the Chinese version of LEE scale and its subscales in these patients with SMI were 0.90 and ranged from 0.86 for “intrusiveness/hostility.” 0.88 for “tolerance,” 0.90 for “emotional Involvement” to 0.92 for “attitude towards patient.” Therefore, the Chinese version demonstrated a good internal consistency and in general, each item was correlated well with its subscale and each subscale also correlated well with the overall scale. All corrected item-total correlations were positive, with all falling between 0.30 and 0.70.

### 9.5. Concurrent Validity of the Chinese Version

The relationships between the LEE scale and the other two theoretically relevant measures and the subscales of one of the two measures (i.e., FAD) are summarized in Table 5. As expected, the total scores of the Chinese version of LEE scale and its four factors were significantly and negatively correlated with the mean scores of FAD (*r* = −0.46, *P* < 0.05 to −0.54, *P* < 0.01) and its subscales (*r* = −0.46, *P* < 0.05 to −0.68, and *P* < 0.001), and significantly and positively correlated with the FBIS (*r* = 0.48, *P* < 0.05 to 0.56, and *P* < 0.01). The total and subscales of the Chinese version of LEE scale were also moderately and positively correlated (*r* = 0.49, *P* < 0.05 to 0.65, and *P* < 0.001).

### 9.6. Reproducibility of the Chinese Version

Mean scores of the Chinese version in patients (*N* = 262) who reported no major changes in both the severity of positive symptoms (eight-item scores of BPRS) and family functioning (FAD scores) between the first and second test over six months were calculated and compared. Intraclass correlation coefficients (ICC) of the LEE scores between the two measurements among those patients (*n* = 100) were 0.90 (*F* = 5.33, df = 98, and *P* = 0.01), indicating a satisfactory level of reproducibility over six months in SMI patients with stable mental state.

### 9.7. Levels of Patients' Perceived Expressed Emotion of Family in SMI and Its Subgroups

In Table 6, levels of perceived EE, family functioning, caregiving burden, and levels of depression and anxiety symptoms were calculated and compared between patients with various psychiatric diagnoses using one-way ANOVA test. Mean total scores (in descending order) of the LEE scale for the patients with SMI in terms of their psychiatric diagnosis were 132.88 (SD = 20.54) for unipolar disorder, 121.47 (SD = 20.33) for psychotic disorders, 119.45 (SD = 23.65) for schizophrenia, and 111.01 (SD = 18.15) for bipolar disorder. The patients with unipolar disorder had the highest mean scores of LEE scale and family functioning (FAD) mean scores, while those with bipolar disorder had the lowest mean scores in all measurements (LEE, family functioning, and caregiving burden (FBIS)).

There were significant differences on the mean scores of LEE total scale and its three subscales (“intrusiveness/hostility”, “attitudes towards patient,” and “emotional involvement”) and family functioning between the illness subgroups (*P* < 0.05). Post hoc Tukey's HSD test results indicated that the level of perceived EE in patients with unipolar disorder was significantly higher than those in the other three illness groups (schizophrenia, psychotic disorders, and bipolar disorder; *P* < 0.01) while the EE levels in schizophrenia and psychotic disorders were also significantly higher than that in bipolar disorder (*P* < 0.03 and <0.01, resp.). For the three subscales, their mean scores in patients with unipolar disorder were significantly higher than those in bipolar disorder (*P* < 0.01), while two of these subscales (“intrusiveness/hostility” and “emotional involvement”) in patients with schizophrenia and psychotic disorders were also significantly higher than that in bipolar disorder (*P* < 0.01). The patients with unipolar disorder also indicated significantly higher family functioning than those with bipolar disorder and in psychotic disorders (both *P* < 0.01). It is also interesting to note that the levels of family burden (FBIS score) in both schizophrenia and psychotic disorders were higher than those in unipolar and bipolar disorders, although they did not statistically differ between the four subgroups.

## 10. Discussion

### 10.1. Satisfactory Psychometric Properties of the Chinese Version of LEE Scale

With few researches on the levels of EE among Chinese and other Asian populations of mental illness, this research serves for not only an examination of the reliability and validity of a Chinese version of the level of expressed emotion (LEE) scale but also providing preliminary assessment of the level of patients' perceived EE in a large sample of 262 Hong Kong Chinese people with different types of severe mental illnesses (SMI). This study also compared the levels of patients' perceived EE between a few major severe mental illnesses, including schizophrenia, psychotic disorders, and unipolar and bipolar mood disorders. First of all, the results of this study indicate that Chinese version of the 52-item LEE scale demonstrated very satisfactory psychometric properties to be considered as a measure of patients' perceived EE of their families in Chinese people with SMI. The Chinese version showed satisfactory to good semantic equivalence in terms of items and the overall scale (kappa values ranged from 0.76–0.95) and the intra-class correlation coefficients of 0.81–0.92 with the original English version. The test-retest reliability over 2-week interval and internal consistency (calculated using the four-factor structure established by confirmatory factor analysis) were high (*r* = 0.89–0.94 and Cronbach's alphas of 0.86–0.92, resp.). The Chinese version demonstrated good concurrent validity with two theoretically relevant measures, indicating strong associations with the valid measures of family functioning (negative relationship) and family caregiving burden (positive relationship) and all of their subscales. Although the family functioning and caregiving burden were found varying across different diagnoses of SMI (e.g., those with unipolar disorder had significantly higher family functioning and those with psychotic disorders had much high family burden than those with both unipolar and bipolar disorders) [32], these significant relationships may reveal the impacts of the patients' perceived EE on their family members' health and well-being in caring for a relative with MI [2, 11]. Therefore, effective strategies in reducing patients' perceived EE may also help these families improve their interpersonal relationships between family members and family harmony and functioning and in turn facilitate family caregivers to cope more effectively with problems and difficulties in caring for both patient and the whole family.

In addition, the Chinese version also indicated a satisfactory reproducibility of the total scores in the SMI patients with stable mental state over a period of six months. These satisfactory results on both reliability and validity and the confirmed factor structure discussed as below support the recommendation by Cole and Kazarian [9] that the LEE scale (and the translated Chinese version in this study) can be a reliable and valid instrument to measure the level of EE of families caring for a relative with SMI, from the patients' perspectives. This self-report scale can be more time efficient and less intensively trained than those for the conventional EE measures (e.g., Camberwell Family Interview and Five-Minute Speech Sample test) and thus can be more easy and convenient to administer in clinical settings. With an increasing emphasis on understanding patients' perceived EE of family members and its impacts on the patients themselves, this validated Chinese version of the LEE scale can be applied to mental health practice for utilizing the promised benefits of understanding and measuring EE, settling the limitations of time constraint and low interest in interviews with family members as suggested by previous studies [3, 4, 12].

### 10.2. Confirmatory Factor Structure of the Chinese Version of LEE Scale

In this study, the hypothesized four-factor structure of the Chinese version of the LEE scale with paths (i.e., correlations between each of the four factors) between all factors was confirmed. This four-factor model was identified from the results of Chien and Chan's [11] study in 321 Chinese people with schizophrenia and similarly in this study, from the exploratory factor analysis among 262 patients with SMI. The four-factor model shows that “intrusive and hostility,” various negative “attitudes toward patient,” level of “tolerance,” and extent of “emotional involvement” are four moderately correlated factors or concepts that cover patients' perceived EE of their families. The four-factor solution of the Chinese version in this study also accounted for a higher percentage of total variance (i.e., >70%) of the perceived EE than that (about 42% and 60%) in Gerlsma et al. [31] and the original authors (Cole and Kazarian) [9] of the LEE scale. Besides explaining more variance, the Chinese version was also shortened (i.e., from the original 60 items to 50 items in this study). The Chinese version in this study can be more convenient and user-friendly to complete a shorter questionnaire and higher construct validity than the original English and other versions, taking account of all the results of validity testing. Furthermore, our findings are similar to those of the pioneer studies of EE by Brown [33] on the key family caregivers' EE using the Camberwell Family Interview that the nature of EE is multidimensional and complex, consisting of four to six domains. The three components of family EE perceived by the patients that are embedded in the LEE scale similar to those identified by Brown [33] and Kuipers et al. [34] included emotional overinvolvement, critical comments/hostility, and positive attitude (or remarks) towards patient.

In addition, the four-factor model of this Chinese version of the LEE scale was also found to be the best fit with the data collected, and thus the construct of patients' perceived EE, when compared with the original two-factor model by Cole and Kazarian [9] and the modified Dutch three-factor model by Gerlsma et al. [31], Intrusiveness/Hostility and Emotional Involvement are the two key components of EE, being most commonly accepted and agreed by the researchers across cultures [15, 35]. The other two factors, including attitudes towards patient and tolerance (of the mental illness and its related behaviors), are more increasingly recognized factors in recent studies, evaluating the emotional climate and interpersonal relationships between family members of people with SMI in Western culture [36]. This perception is also consistent with the Chinese belief that open expression of emotions and comments, either positive or negative, is not preferred and encouraged and thus self control of emotions and negative remarks are being highly emphasized [11, 37]. There is much research evidence suggesting that negative attitudes and intolerance towards other people's behaviors in Chinese culture, together with excessive emotions, are harmful to one's own physical and psychological health [12], which may explain the reasons for not only emotional involvement but also negative attitudes and/or tolerance to be treated as the other two major components of perceived EE of their families in this study. A member in a Chinese family should be expected to keep his/her emotions under control to maintain family functioning and relationships or otherwise, he/she would be considered unfavorable or nonpreferable to other family member's mental well-being, thus attributing to a higher level of perceived family EE. The importance of these two factors, including attitude and tolerance towards patient is also consistent with the findings of a few Western studies [31, 38]. Butzlaff and Hooley [4] suggested that family members with high EE are critical and negative towards patient's behaviors, and often expect the patient to take main responsibility for and be able to control his/her emotions and illness-related behaviors. The findings on the four-factor structure provide a further support for the proposed multi-dimensional nature of the family attitude and emotional environment in caring for a patient with SMI, as suggested by the original authors of the LEE scale and a few recent studies [9, 38, 39]. The construct and factor structure of perceived EE of family in the translated Chinese version of LEE scale should be further investigated in Chinese people with SMI and its subtypes, as well as with diverse sociodemographic and clinical backgrounds.

### 10.3. Levels of Perceived EE in Chinese Patients with SMI

From the mean scores of the Chinese version of LEE scale in this study, the participants in all the subgroups of SMI reported a moderate level of perceived EE (from 111.01 ± 18.15 to 132.88 ± 20.54; possible score range 50–200). The patients with unipolar disorder expressed significantly higher perceived EE of their family members than those with the other three illness subgroups (schizophrenia, psychotic disorders, and bipolar disorder), in terms of the mean total score and most of the four subscales, and those with bipolar disorder reported the relatively lowest mean total and subscale scores among the four subgroups of SMI. In addition, the patients with psychotic disorders also indicated significantly higher perceived EE than those with bipolar disorder. These findings provide evidence that the Chinese depressed patients had the highest level of perceived EE of the family among the major subgroups of SMI. Similar to the findings of Gerlsma et al. [31] and Hooley and Teasdale [39], the depressed patients indicated high levels of perceived EE, mainly relating to their high perceived intrusiveness and irritability (emotion overinvolvement) and inadequate social support obtained from their family members. Therefore, the development and course of depression can be seen as a dynamic interactional process in which family support and caring attitude can serve as a buffer of the onset of the illness or a mediator of the recovery process. In addition, if support-seeking of the depressed patients meet with negative responses such as criticisms and neglects from their family members, they are likely to perceive such detrimental responses as the “true” reflection of the emotional climate of their family (i.e., high level of perceived EE in this study). Hence, they would react with negative thoughts and exaggerated failure that they are being rejected by their family and end up with a high risk of relapse [40, 41].

When compared to Ng and Sun's [35] study in 202 Chinese patients with schizophrenia in Hong Kong and Macao using the concise version of this Chinese LEE scale, the mean values of the total score and two subscales (intrusiveness/hostility and emotional involvement) among the patients with schizophrenia and psychotic disorders in this study are slightly higher than the adjusted mean values of total score reported by their participants (i.e., 119.45 (schizophrenia) and 121.47 (psychotic disorders) versus 112.67), and of two subscales (30.43 and 32.24 versus 30.17 for subscale “intrusiveness/hostility”; 33.54 and 35.77 versus 32.13 for subscale “emotional involvement”). Among only a few studies using the LEE scale, Donat [42] measured the perceived EE among 188 patients with SMI in the USA and reported that the mean values (adjusted to 50 items) of the total and all subscale scores were about 104.50 and from 24.2 to 29.8, respectively. In contrast to the findings of Azhar and Varma [41] and Ikram et al. [43] that majority of families (45–75%) in few Asian and Western countries reported a low level of EE, most of the Chinese patients in this study indicated an average of moderate to high levels of perceived EE. On the other hand, the findings in this study echoed those in Li and Authur's [12] and Philip et al.'s [14] studies that over 40% of family members of patients with schizophrenia were rated as having high EE.

However, when compared to another study conducted by Gerlsma and Hale III [2] in 26 depressed outpatients using the Dutch version of LEE scale, the mean values of the total score and two subscales (intrusiveness/hostility and emotional involvement) among the patients with unipolar disorder in this study are much higher than the adjusted mean values reported by their participants (i.e., mean total score of 132.86 versus 105.43, mean “intrusiveness/hostility” score of 30.52 versus 28.12, and mean “emotional involvement” score of 34.98 versus 33.70, resp.). The results of these comparisons further illustrate that it is highly possible for patients' perceived expressed emotion of their family to vary much across cultures, by how a culture defines family life and relationships and what behavior patterns are considered appropriate for familial and other social interactions. Therefore, attitude and emotional responses to a mentally ill relative such as protection, hostility, anger and devotion may vary according to family dynamics and practices within one's specific cultural context [37, 43]. In addition, Chinese patients with SMI, especially those with unipolar disorder (depression), perceived much higher levels of intrusiveness, hostility and emotional involvement of family members towards them than those in a few Western and Asian countries. This may also highlight the uniqueness of these two (patients' perceived) family's emotional responses towards their mentally ill relative in Hong Kong Chinese culture. Consistent with the results of this study, these families may also perceive higher levels of burden of care. For instance, a high degree of collectiveness and involvement in family affairs by family members or caregivers is a traditional and common behavior pattern among Chinese families. Therefore, the interpretation of the dimension and degree of EE may require the inclusion of different cultures in order to be valid and accurate.

The translated Chinese version of the LEE scale has demonstrated sound psychometric properties and thus can be applied to mental health practice for better understanding and measuring the levels of EE among Chinese psychiatric patient populations. This self-report Chinese version of LEE scale has the advantages that they are easily conducted, allow for repeated measurements, require minimal training and relatively more objective interpretation, and allow for the clients' own perception. However, the results of such self-reporting should be confirmed by data from direct behavioral observations about family interactions and activities, which are considered highly relating to the development and course of mental illness. While not substituting for direct family observations, the LEE scale tested in this study can be a helpful complementary measuring tool of EE for both researchers and clinicians. When considering time constraints and intensive training required for the conventional family interviews, this LEE scale can provide a user-friendly, brief and readily applicable instrument, which appears to be reliable and convenient to be administered in contemporary community mental healthcare settings.

A universal model of the construct of EE and its relationships with and effects on patients with SMI should consist of not only the previous elements suggested by Brown et al. in 1960s and Vaughn and Leff in 1970s but also the variation and influence of cultures [1, 3]. Further testing of this translated Chinese version of LEE scale in patient populations with diverse sociocultural backgrounds is recommended before it is widely used in different clinical settings. With the results of satisfactory psychometric properties of this Chinese version, this LEE scale can be further tested in and applied to the healthy sample and general public, as well as different Chinese communities.

### 10.4. Limitations of the Study

There were a few limitations of this research for instrument validation. First, this study only used the SMI patients' self-reports of their perceived EE. In contrast with the conventional family members' interviews, it could be possible that the responses or ratings from the patients with SMI are unreliable due to the effects of illness symptoms and the correlations of the LEE and other psychosocial and mental health measures would be artificially inflated. While some researchers have argued that family caregivers are better reporters of their attitude and emotional climate toward the patient than the patients themselves, Hooley and Teasdale [39] indicated that the patients' perception of the criticism and emotional responses they had received was more predictive of their relapse than the amount of criticism actually expressed by the family members during the interview. Similar to the data collection procedure used in this study, the researchers need to ensure a high level of reliability of the patients' self-reports by checking their mental stability and competence of participation in the research and completion of the self-report questionnaires.

Second, the sample in this study was selective. Most of the participants were male, well educated, Hong Kong born Chinese, mentally stable, with primary diagnosis of schizophrenia and psychotic disorders and with no comorbidity of any other mental illnesses. The participants were recruited only from one psychiatric outpatient clinic in Hong Kong, where similar socio-economic backgrounds and mental healthcare services were found. The family caregivers were mainly well educated and middle class people and highly motivated to participate in this study. In addition, the sample size was also relatively small for a factor analysis of a 52-item scale. Therefore, these results are in need of replication in various kinds of psychiatric patients with more diverse sociodemographic and clinical backgrounds.

Third, the findings were by no means clear on how the Chinese version of LEE scale could be related to the original EE concept, which is operationally defined to measure the emotional climate and stress environment in a family from the perspective of family caregivers. Despite the fact that previous comparisons had been made, reexamining the convergent validity of the Chinese version of LEE scale with the standard measures such as the Camberwell Family Interview schedule could further evidence whether the Chinese version would confirm whether both parties had similar perceptions of family's EE.

## 11. Conclusions

The findings of this study provide evidence to support that the translated 50-item Chinese version of LEE scale is reliable and valid in measuring patients' perceived EE of family members among Chinese people with SMI, including schizophrenia and its subtypes and mood disorders. In addition to indicating very satisfactory reliabilities and concurrent validity, the Chinese version also showed a four-factor structure accounting for a high percentage (about 72%) of the total variance of the EE construct in exploratory factor analysis and pointing to the best fit with the data with medium or medium-large associations between all factors and their corresponding items and thus the EE construct. Finally, most of the participants with SMI reported a moderate level of perceived EE and those with unipolar disorder expressed significantly higher perceived EE of their family members than those with the other three illness subgroups (schizophrenia, psychotic disorders, and bipolar disorder). The high mean scores of subscales “intrusiveness/hostility” and “emotional involvement” in these patients, particularly in those with unipolar disorder (depression), raise our particular attention of these two domains in the assessment of EE among Chinese patients with SMI and design of family-centered interventions for these patients.

## Figures and Tables

**Table 1:** Characteristics of patients with severe mental illness and nonrespondents.

Characteristics	Respondents (*n* = 262) *F* (%)	Those refusals (*n* = 59) *F* (%)	Chi-square or *t* test value
Gender			χ^2^ = 2.08
Female	102 (38.9)	22 (37.3)	
Male	160 (61.1)	37 (62.7)	
Education level			*χ* ^2^ = 2.19
Primary school or below	30 (11.5)	6 (10.2)	
Secondary school	181 (69.1)	42 (71.2)
Tertiary (e.g., university or postgraduate study)	51 (19.5)	11 (18.6)
Primary psychiatric diagnosis			*χ* ^2^ = 1.58
Bipolar affective disorders	12 (4.6)	3 (5.1)	
Psychotic disorders	50 (19.1)	13 (22.0)	
Schizophrenia	118 (45.0)	26 (44.1)	
Unipolar affective disorders (e.g., major depression)	48 (18.3)	10 (16.9)	
Others (e.g., personality disorders and dual diagnoses)	34 (13.0)	7 (11.9)	
Psychiatric medications			χ^2^ = 1.58
Antidepressants	50 (19.1)	11 (18.6)	
Anticonvulsants	7 (2.7)	2 (3.4)	
Atypical antipsychotics	90 (34.4)	19 (32.2)	
Conventional antipsychotics	83 (31.7)	19 (32.2)	
Lithium salts	6 (2.3)	1 (1.7)	
Both anti-depressants and antipsychotics	20 (7.6)	4 (6.8)	
Psychiatric treatments receiving			χ^2^ = 1.48
CPN visits and education	178 (67.9)	30 (50.8)	
Family therapy/education	32 (12.2)	8 (13.6)	
Medication compliance management	102 (38.9)	16 (27.1)	
Psychoeducation	98 (37.4)	28 (47.5)	
Social and work skills training	87 (33.2)	19 (32.2)	
Others (e.g., relaxation and self-regulation)	75 (28.6)	20 (33.9)	

	M ± SD, range	M ± SD, range	

Age (years)	29.12 ± 10.05, 19–45	29.45 ± 8.91, 20–46	*t* = 1.12
Duration of mental illness (months)	35.21 ± 14.25, 12–98	32.90 ± 17.02, 14–96	*t* = 1.98
Rehospitalization in the past 3 months			
Number of readmissions	0.40 ± 0.29	0.49 ± 0.31	*t* = 3.38*
Length of rehospitalizations (days)	8.12 ± 4.11	10.01 ± 6.38	*t* = 2.16
Number of family members living with patient	2.25 ± 0.98, 1–5	2.13 ± 0.98, 1–4	*t* = 1.31
Average contact time with main caregiver (hours/week)	30.40 ± 9.54, 8–44	29.13 ± 11.49, 7–30	*t* = 1.04

*P < 0.05.

**Table 2:** Characteristics of family caregivers (*N* = 262).

Characteristics	*F* (%)
Gender	
Female	158 (60.3)
Male	104 (39.7)
Age (years), M ± SD (range)	42.58 ± 10.82 (range 21–67)
Education level	
Primary school or below	48 (18.3)
Secondary school	182 (69.5)
Tertiary (e.g., university or postgraduate study)	32 (12.2)
Relationship with patient	
Child	38 (14.5)
Parent	98 (37.4)
Sibling	33 (12.6)
Spouse	63 (24.0)
Others (e.g., grandparent and nephew)	30 (12.5)
Household income, monthly (HKD)	
5,000 or below	8 (3.1)
5,001–10,000	43 (16.4)
10,001–20,000	103 (39.3)
20,001–30,000	83 (31.7)
More than 30,000	25 (9.5)

US$1 = HK dollars 7.8.

**Table 3:** Results of varimax rotation of four factors identified from the Chinese version.

Items	Factor loading			
	Factor 1	Factor 2	Factor 3	Factor 4
(1) Does not butt into my conversations (3)^a^	0.49			
(2) Is not overprotective with me (6)	0.47			
(3) Does not insist on doing things with me (14)	0.48			
(4) Does not pry into my life (41)	0.47			
(5) Supports me when I need it (36)	0.56			
(6) Is not always interfering (10)	0.46			
(7) Leaves me feeling overwhelmed (20)	0.49			
(8) Often checks up me to see what I am doing (24)	0.46			
(9) Isn't always nosing into my business (28)	0.51			
(10) Always has to know everything about me (32)	0.49			
(11) Butts into my private matters (37)	0.45			
(12) Gets upset when I do not check in with him/her (49)	0.52			

(1) Is sympathetic toward me when I'm ill or upset (8)		0.51		
(2) Encourages me to seek outside help when I'm not feeling well (12)		0.48		
(3) Makes me feel valuable as a person (19)		0.50		
(4) Tries to make me feel better when I'm upset or ill (26)		0.50		
(5) Is willing to gain more information to understand my condition when I'm not feeling well (39)		0.42		
(6) Doesn't blame me when I'm feeling unwell (43)		0.47		
(7) Tries to reassure me when I'm not feeling well (51)		0.41		
(8) Says I just want attention when I say I'm not well (4)		0.45		
(9) Doesn't help me when I'm upset or feeling unwell (15)		0.47		
(10) Says I cause my troubles to occur in order to get back at him/her (22)		0.50		
(11) Says it is OK to seek professional help (30)		0.45		
(12) Accuses me of exaggerating when I say I'm unwell (34)		0.50		
(13) Often accuses me of making things up when I'm not feeling well (47)		0.48		

(1) Is tolerant with me even when I'm not meeting his/her expectations (2)			0.45	
(2) Can see my point of view (9)			0.41	
(3) Doesn't feel that I'm causing him/her a lot of trouble (13)			0.43	
(4) Understands my limitations (23)			0.46	
(5) Blames me for things not going well (18)			0.51	
(6) Is realistic about what I can and cannot do (27)			0.49	
(7) Is understanding if I make mistakes (40)			0.42	
(8) Makes me feel guilty for not meeting his/her expectations (5)			0.41	
(9) Puts me down if I don't live up to his/her expectations (16)			0.40	
(10) Gets angry with me when things don't go right (31)			0.42	
(11) Is impatient with me when I'm not well (42)			0.42	
(12) Hears me out (29)			0.43	

(1) Calms me down when I'm upset (1)				0.41
(2) Doesn't panic when things start going wrong (11)				0.40
(3) Is able to be in control in stressful situations (25)				0.43
(4) “Flies off the handle” when I don't do something well (48)				0.42
(5) Makes me feel relaxed when he/she is around (33)				0.41
(6) Can cope well with stress (38)			0.40	0.45
(7) Loses his/her temper when I'm ill or upset (7)				0.41
(8) Doesn't insist on being with me all the time (17)				0.41
(9) Doesn't know how to handle my feelings when I'm not feeling well (21)				0.45
(10) Gets angry with me for no reason (35)				0.42
(11) Expects too much from me (44)				0.43
(12) Makes matters worse when things are not going well (46)				0.41
(13) Gets irritated when things do not go right (50)				0.43

Percentage of variance explained	21.48	19.32	16.01	14.98

^a^The item number of the original version of the LEE scale.

Factor loadings ≥ 0.40 are reported.

LEE: level of expressed emotion scale.

Factor 1: Intrusiveness/hostility; Factor 2: attitude towards patient; Factor 3: tolerance; Factor 4: emotional involvement.

**Table 4:** Summary of fit indices of three-hypothesized models of LEE scale (*N* = 262).

Model	*χ* ^2^	df	*χ* ^2^/df	*P* value	GFI	AGFI	TLI	RMSEA (90% CI)	SRMR	WRMR
Model 1										
Uncorrelated factors	98.34	50	1.97	0.80	0.97	0.96	1.00	0.040 (0.036–0.044)	0.039	0.85
Correlated factors^a^	92.58	48	1.93	0.75	0.99	0.98	1.02	0.031 (0.027–0.035)	0.028	0.78
Model 2										
Uncorrelated factors	102.33	50	2.05	0.54	0.88	0.87	0.89	0.052 (0.044–0.060)	0.050	0.92
Correlated factors	97.02	48	2.02	0.58	0.89	0.90	0.95	0.050 (0.042–0.058)	0.054	0.89
Model 3										
Uncorrelated factors	134.21	50	2.68	0.20	0.86	0.85	0.89	0.071 (0.061–0.081)	0.071	0.99
** **Correlated factors	125.88	48	2.60	0.25	0.89	0.88	0.91	0.067 (0.055–0.076)	0.060	0.94

Model 1: four-factor model identified by Li and Arthur [12] and in this research, Model 2: two-factor model suggested by Startup in 1999 [10], and Model 3: three-factor model suggested by Kim and Miklowitz in 2004 [32].

^a^Model fit indices tested with paths (correlations) set-up between the hypothesized factors in each model.

*χ* ^2^: Chi-squared goodness of fit; df: degree of freedom; *P* value (a good fit if *P*≧0.1); GFI: goodness-of-fit index (ranging from 0 to 1, a good fit if GFI≧0.9); AGFI: adjusted goodness of fit index (similar to GFI, a good fit if AGFI≧0.9); TLI: Tucker-Lewis index (0.90–0.95 acceptable, a good fit if TLI > 0.95); RMSEA: root mean square error of approximation (a good fit if RMSEA≦0.05); SRMR: standardized root mean square residual, (a good fit if SRMR < 0.05); WRMR: weighted root mean residual (a good fit if WRMR < 0.90).

**Table 5:** Pearson's correlations between the LEE scale and other theoretically relevant measures (*N* = 262).

Measures	LEE	IN/H	AP	TO	EI	FAD	CO	AR	AI	BC
LEE	1.00									
Intrusiveness/Hostility	0.584**	1.00								
Attitudes towards patient	0.542**	0.543**	1.00							
Tolerance	0.521**	0.524**	0.592**	1.00						
Emotional involvement	0.645***	0.569**	0.553**	0.485*	1.00					
FAD	−0.538**	−0.578**	−0.456*	−0.502**	−0.507**	1.00				
Communication	−0.540**	−0.506**	−0.533**	−0.498**	−0.461*	0.471*	1.00			
Affective responsiveness	−0.503**	−0.532**	−0.602***	−0.528**	−0.610**	0.518**	0.523**	1.00		
Affective involvement	−0.541**	−0.554**	−0.557**	−0.556**	−0.679***	0.551**	0.562**	0.619***	1.00	
Behavioral control	−0483*	−0.601**	−0.581**	−0.485*	−0.481*	0.538**	0.498**	0.491**	0.467*	1.00
FBIS	0.561**	0.564**	0.533**	0.504**	0.475*	−0.517**	−0.495**	−0.512**	−0.512**	−0.461*

LEE: level of expressed emotion scale, LEE subscales: IN/H: intrusiveness/hostility, AP: attitudes towards patient; TO: tolerance; and EI: emotional involvement.

FAD: family assessment device, Its subscales, CO: communication; AR: affective responsiveness; AI: affective involvement; and BC: behavioral control.

FBIS: family burden interview schedule.

*P < 0.05;**P < 0.01; ***P < 0.001.

**Table 6:** Levels of perceived expressed emotion and other psychological measures in patients with different psychiatric diagnoses.

Instrument	Schizophrenia *n* = 118 M ± SD	Psychotic disorders *n* = 50 M ± SD	Unipolar disorder *n* = 48 M ± SD	Bipolar disorder *n* = 12 M ± SD	One-way ANOVA test *F*, df, and *P*
LEE (50–200)^a^	119.45 ± 23.65	121.47 ± 20.33	132.88 ± 20.54	111.01 ± 18.15	5.21, 259, 0.01
Intrusiveness/hostility (12–48)	30.43 ± 10.10	32.24 ± 10.87	30.52 ± 10.10	26.38 ± 9.80	5.98, 258, 0.005
Attitudes towards patient (13–52)	28.87 ± 9.89	26.69 ± 10.76	30.88 ± 9.12	27.54 ± 9.38	4.57, 258, 0.04
Tolerance (12–48)	26.61 ± 11.41	26.77 ± 11.89	27.50 ± 10.34	26.32 ± 10.02	3.83, 258, 0.12
Emotional involvement (13–52)	33.54 ± 10.06	35.77 ± 10.57	34.98 ± 12.11	30.77 ± 10.80	5.17, 258, 0.01
BPRS-positive symptoms^b^ (0–48)	24.98 ± 8.57	25.33 ± 9.81	15.51 ± 6.40	18.01 ± 8.11	6.10, 258, 0.005
BDI (0–63)	18.21 ± 8.90	19.01 ± 7.88	22.21 ± 9.98	21.65 ± 9.65	4.33, 258, 0.05
BAI (0–63)	27.91 ± 9.10	29.05 ± 10.54	30.56 ± 11.03	31.01 ± 10.51	3.45, 258, 0.15
FAD (60–240)	154.98 ± 23.67	145.33 ± 24.79	177.30 ± 19.12	149.58 ± 24.01	5.10, 259, 0.01
FBIS (0–50)	10.75 ± 6.02	10.69 ± 6.59	10.17 ± 6.85	10.01 ± 7.08	3.95, 259, 0.08

LEE: level of expression emotion scale; BPRS: brief psychiatric rating scale; BDI: beck depression inventory-II; BAI: beck anxiety inventory; FAD: family assessment device; FBIS: family burden interview schedule.

^a^Possible range of scores of the total scale or its subscales in the parentheses.

^b^Mean score calculated with 8 items of the BPRS from thought disturbance and disorganization subscales [26].
